# Successful elimination of non–pulmonary vein foci by pulsed-field ablation in a patient with persistent atrial fibrillation: A case report

**DOI:** 10.1016/j.hrcr.2025.08.035

**Published:** 2025-09-05

**Authors:** Shin Hasegawa, Taiji Miyake, Yoriaki Shinoda, Mutsumi Aoyama, Nana Nishikawa, Hitoshi Matsuo

**Affiliations:** 1 Department of Cardiology, Gifu Heart Center, Gifu City, Gifu, Japan; 2 Department of Clinical Engineering, Gifu Heart Center, Gifu City, Gifu, Japan

**Keywords:** Atrial fibrillation, Complex signal identification, Left atrial septum, Non–pulmonary vein trigger, Premature atrial contractions, Pulsed-field ablation

**Key Teaching Points**

• Incessant atrial fibrillation occurred immediately after pulmonary vein isolation (PVI) and posterior wall isolation using pulsed-field ablation (PFA), triggered by frequent premature atrial contractions (PACs). These PACs were identified as originating from the left atrial septum using complex signal identification and were successfully eliminated with additional PFA applications.
• This case highlights the potential of PFA to effectively target non–pulmonary vein foci, extending its utility beyond conventional PVI.
• However, the appearance of new low-voltage areas in the right atrium after left atrial septal ablation suggests a risk of excessive transmural lesion formation, emphasizing the need for careful energy delivery during septal ablation.

## Introduction

Pulmonary vein isolation (PVI) is the cornerstone of catheter ablation for persistent atrial fibrillation (AF). However, maintenance of sinus rhythm (SR) can be challenging because of the presence of non–pulmonary vein (non-PV) foci.^1^

Pulsed-field ablation (PFA) is an innovative energy modality for PVI and has been shown to be noninferior to radiofrequency and cryoablation, with the potential to create more durable lesions.^2^^,^^3^ Although the implementation of PFA is expanding rapidly, its safety and efficacy for non-PV foci have not been fully evaluated.^1^

We report a case in which PVI and posterior wall isolation (PWI) using PFA were performed in a patient with persistent AF. Immediately after isolation, frequent premature atrial contractions (PACs) originating from the septum induced and sustained AF. Additional PFA was delivered to high-frequency fragmented potentials identified in the left atrial (LA) septum, leading to successful elimination of the arrhythmogenic activity.

## Case report

A 74-year-old woman with dyslipidemia presented with palpitations. She was diagnosed with long-standing persistent AF of 2 years’ duration. As her arrhythmia was refractory to pharmacological therapy, she provided informed consent to undergo catheter ablation, including PVI and PWI.

The procedure was performed under deep sedation. Preprocedural contrast-enhanced computed tomography revealed LA enlargement (83 mL/m^2^) without thrombus. Unfractionated heparin was administered to maintain an activated clotting time of 350–400 seconds. A 3-dimensional electroanatomic map was created using the CARTO system (Biosense Webster), and ablation was performed during AF. After femoral and internal jugular venous access, a 20-electrode atrial cardioversion catheter (BeeAT, Japan Lifeline) was placed in the coronary sinus. A single transseptal puncture was performed under intracardiac echocardiographic and fluoroscopic guidance. A FARADRIVE sheath (Boston Scientific) was introduced, and PFA was performed using the FARAPULSE system (Boston Scientific).

**Figure 1:**
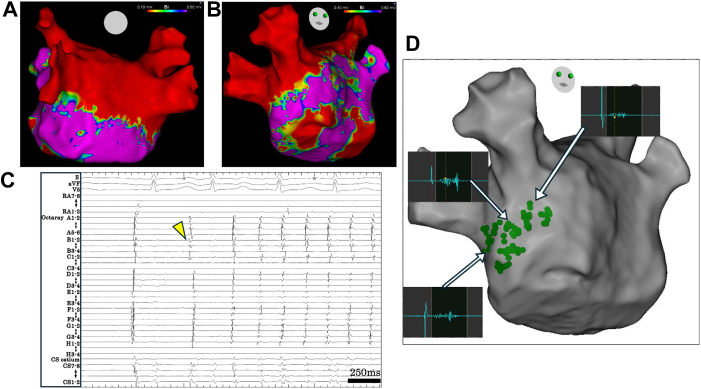
**A and B:** Bipolar voltage map of the left atrium after PVI and PWI using PFA. **C:** Intracardiac electrogram demonstrating a PAC originating from the left atrial septum (*yellow arrow*), which triggered atrial fibrillation. **D:** CSI map derived from the bipolar voltage map, highlighting areas with fractionated signals. *Green tags* indicate sites with a CSI score of 10. Local electrograms at these sites exhibited prolonged fractionated potentials, with the longest duration reaching 126 ms. CSI = complex signal identification; PAC = premature atrial contraction; PFA = pulsed-field ablation; PVI = pulmonary vein isolation; PWI = posterior wall isolation.

PVI was achieved with 8 applications per vein (2 basket pairs and 2 flower pairs, each rotated 45° between applications). PWI was completed with 6 roofline and 8 bottom-line applications. SR was restored during the procedure. A voltage map obtained during constant right atrial (RA) pacing confirmed complete isolation of the PVs and posterior wall (Figure 1A and 1B). However, upon cessation of pacing, frequent PACs triggered recurrent AF (Figure 1C). Although cardioversion briefly restored SR, AF was reproducibly reinduced by the PACs, prompting additional intervention.

**Figure 2:**
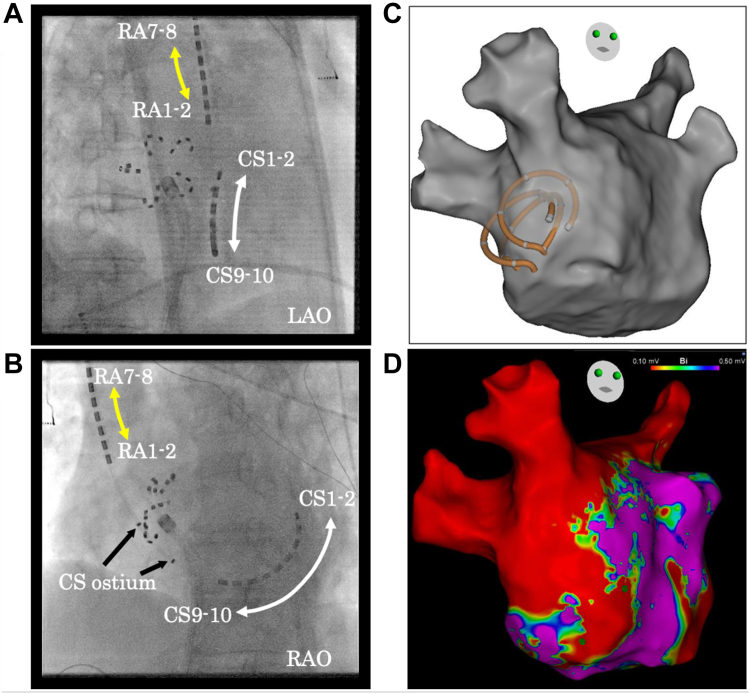
**A and B:** Fluoroscopic images showing the Farawave catheter positioned at the left atrial septum. A BeeAT catheter is positioned in the RA (*yellow and black arrows*) and the CS (*white arrow*). **C:** CARTO image displaying the Farawave catheter in a ring configuration at the left atrial septum. Three applications were delivered at this site. **D:** Bipolar voltage map of the left atrium after additional PFA applications to the septum. CS = coronary sinus; LAO = left anterior oblique; PFA = pulsed-field ablation; RA = right atrium; RAO = right anterior oblique.

Because of sustained AF, mapping of the PACs was not feasible. Voltage mapping during RA pacing identified complex signal identification (CSI) areas in the LA septum (Figure 1D). Placement of the OCTARAY catheter (Biosense Webster) in the high CSI-score area revealed the earliest activation site of the PACs, corresponding to the highest CSI-score area (Figure 2A and 2B). Three additional PFA applications in a flower configuration at this site eliminated the PACs (Figure 2C; Supplemental Videos 1 and 2).

**Figure 3:**
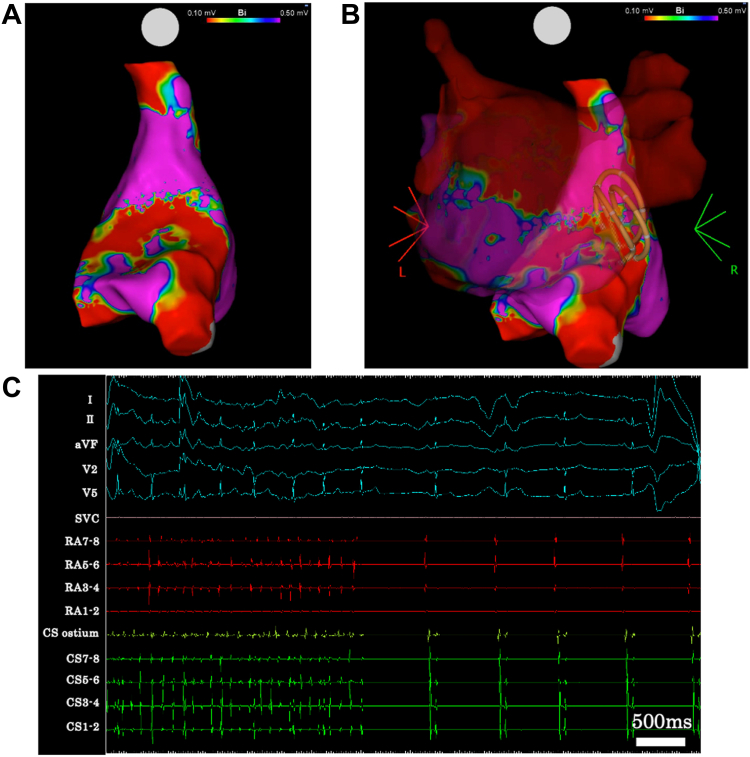
**A:** Bipolar voltage map of the right atrium after septal ablation. **B:** Fusion image of the left atrium and the additional ablation sites. The Farawave catheter is in the flower configuration and appears as a ring on the CARTO map. **C:** Intracardiac electrogram showing spontaneous termination of atrial fibrillation after septal ablation. The surface electrocardiogram shows a normal PQ interval.

Voltage mapping during SR after ablation confirmed low-voltage areas in the LA CSI region and the corresponding septal site of the RA (Figures 2D, 3A, and 3B). AF was briefly reinduced by isoproterenol infusion and programmed stimulation but terminated spontaneously (Figure 3C). The maximum heart rate during isoproterenol administration was 118 beats/min, and no sinus pauses suggestive of sinus node dysfunction were observed throughout the procedure. The PQ interval was 166 ms before the additional applications and 164 ms afterward, indicating preserved atrioventricular conduction. The patient did not complain of palpitations suggestive of PACs or AF, and no recurrence was observed on follow-up 12-lead electrocardiography.

## Discussion

PFA delivers high-voltage, short-duration pulses that create irreversible membrane pores, resulting in cell death and fibrosis through a nonthermal mechanism.^4^ Clinical studies report arrhythmia-free rates comparable to thermal energy ablation for AF.^1^^,^^5^^,^^6^ Because of its cardioselectivity and ability to form transmural lesions without damaging adjacent tissues, PFA has demonstrated a favorable safety profile, supporting its expanding clinical use.^1^^,^^7^ Although PVI remains the foundation of persistent AF ablation, the complexity of the arrhythmogenic substrate often necessitates adjunctive ablation strategies.^1^ In this case, voltage mapping after PVI and PWI revealed fragmented potentials adjacent to the ablation lesion in the LA septum, with an amplitude of 0.08 mV and a duration of 126 ms. Multiple high-frequency potentials were observed near the ablation lines.

Typically, double potentials consisting of far-field and near-field components were observed in the atrial septum, suggesting activation from anatomically distinct regions. However, true conduction between the RA and the LA is uncommon because of functional conduction block, despite histological continuity at the fossa ovalis.^8^ The observed potentials were not typical double potentials but rather fragmented electrograms at the anteroseptal junction adjacent to the right PV antrum.

CSI is a feature of CARTO version 8 that quantitatively evaluates local potential fractionation. Originally developed for atrial flutter, CSI has been expanded to identify complex electrograms and target non-PV foci, a method termed *complex signal identification for fractionated signal area in the atrial muscle*.^9^^,^^10^ Fractionation scores range from 0 to 10, based on parameters such as duration, amplitude, and activation time. In this case, a high CSI score facilitated identification of the PAC origin in the septum.

The septum is recognized as a potential origin of PACs and atrial tachycardia. Compared with radiofrequency ablation, PFA creates more uniform lesions with fewer residual potentials at lesion borders.^1^^,^^8^ Thus, the non-PV foci in the septum were presumed to have preexisted the PVI and to have contributed to the maintenance of the AF substrate. In this case, electrical isolation of the interatrial septum alone was deemed insufficient, and additional substrate modification targeting the presumed origin of the PACs was considered necessary. Given the potential limitations in maneuverability associated with conventional radiofrequency ablation catheters—particularly in achieving stable tissue contact and creating durable lesions—energy delivery was performed using the Farawave catheter. To ensure adequate contact with the atrial septum, the catheter was deployed in a flower configuration, and contact was confirmed angiographically before energy delivery (Supplemental Videos 1 and 2). Because the CSI region was relatively extensive, 3 applications were required to achieve adequate substrate modification. Li et al^11^ and Mohanty et al^12^ emphasized the importance of targeting both anatomical and electrophysiological substrates for optimal outcomes in persistent AF. In this case, AF spontaneously terminated immediately after ablation of the abnormal septal potentials. Termination of AF during the procedure may be associated with improved long-term outcomes.^11^ Although PFA has been reported to be effective for atrial tachycardia originating from LA septal aneurysms, this case suggests that its utility extends to AF substrates as well.^13^ The atrial septum may serve as a viable target for PFA in addressing non-PV foci. Although the procedure was performed safely in this case, larger prospective studies are needed.

This study has limitations. Ablation in the LA septum resulted in transmural lesions extending into the RA. Although PFA reliably creates uniform lesions, septal ablation may produce excessive modification. Matsuura et al^14^ reported that newly developed low-voltage areas in the superior vena cava—defined as regions with amplitudes <0.5 mV and surface area ≥0.5 cm^2^—were observed in 82.4% of patients after PFA and that 14.7% exhibited circumferential involvement of the superior vena cava.

Therefore, caution is advised in patients at risk of sinus node dysfunction or atrioventricular block.^15^ Moreover, septal targeting via LA with the FARAPULSE system may be challenging in certain anatomies.^13^ Although PFA has been associated with less chronic fibrosis and preserved local myocardial function compared with thermal ablation, concerns remain regarding proarrhythmic remodeling, long-term LA function, and thromboembolic risk.^1^

## Conclusion

Additional PFA targeting triggered PACs after PVI and PWI effectively eliminated arrhythmogenic activity in the LA septum. These findings suggest the potential efficacy of PFA for non-PV foci beyond the PVs.

## Declaration of generative AI and AI-assisted technologies in the writing process

During the preparation of this work the authors used a generative AI tool (ChatGPT) in order to assist with language editing and formatting. After using this tool/service, the author(s) reviewed and edited the content as needed and take(s) full responsibility for the content of the publication.

## Disclosures

The authors have no conflicts of interest to disclose.
