# Supplementary material for: Successful elimination of non–pulmonary vein foci by pulsed-field ablation in a patient with persistent atrial fibrillation: A case report
Source: HeartRhythm Case Rep. 2025 Sep 5;11(12):1288–92. doi: 10.1016/j.hrcr.2025.08.035 (PMC12805274; doi:10.1016/j.hrcr.2025.08.035)
Supplement: Supplementary Material [file mmc3.docx]

**Supplementary Video Legends**

**Supplementary Video 1.**PFA Application on Atrial Septum (LAO view)
The Farawave catheter was positioned in contact with the atrial septum, and pulsed field ablation was applied under fluoroscopic guidance in the left anterior oblique (LAO) view.

**Supplementary Video 2.**PFA Application on Atrial Septum (RAO view)
The Farawave catheter was positioned in contact with the atrial septum, and pulsed field ablation was applied under fluoroscopic guidance in the right anterior oblique (RAO) view.
